# Clofazimine for Treatment of Cryptosporidiosis in Human Immunodeficiency Virus Infected Adults: An Experimental Medicine, Randomized, Double-blind, Placebo-controlled Phase 2a Trial

**DOI:** 10.1093/cid/ciaa421

**Published:** 2020-04-11

**Authors:** Py Iroh Tam, S L M Arnold, L K Barrett, C R Chen, T M Conrad, E Douglas, M A Gordon, D Hebert, M Henrion, D Hermann, B Hollingsworth, E Houpt, K C Jere, R Lindblad, M S Love, L Makhaza, C W McNamara, W Nedi, J Nyirenda, D J Operario, J Phulusa, G V Quinnan, L A Sawyer, H Thole, N Toto, A Winter, W C Van Voorhis

**Affiliations:** 1 Paediatrics and Child Health Research Group, Malawi-Liverpool Wellcome Trust Clinical Research Programme, Blantyre, Malawi; 2 Liverpool School of Tropical Medicine, Liverpool, United Kingdom; 3 Center for Emerging and Re-emerging Infectious Diseases, University of Washington, Seattle, Washington, USA; 4 Emmes, Rockville, Maryland, USA; 5 Institute of Infection and Global Health, University of Liverpool, Liverpool, United Kingdom; 6 Bill & Melinda Gates Foundation, Seattle, Washington, USA; 7 Division of Infectious Diseases and International Health, University of Virginia, Charlottesville, Virginia, USA; 8 Calibr, La Jolla, California, USA

**Keywords:** cryptosporidium, diarrhea, HIV, therapeutic, trial

## Abstract

**Background:**

We evaluated the efficacy, pharmacokinetics (PK), and safety of clofazimine (CFZ) in patients living with human immunodeficiency virus (HIV) with cryptosporidiosis.

**Methods:**

We performed a randomized, double-blind, placebo-controlled study. Primary outcomes in part A were reduction in Cryptosporidium shedding, safety, and PK. Primary analysis was according to protocol (ATP). Part B of the study compared CFZ PK in matched individuals living with HIV without cryptosporidiosis.

**Results:**

Twenty part A and 10 part B participants completed the study ATP. Almost all part A participants had high viral loads and low CD4 counts, consistent with failure of antiretroviral (ARV) therapy. At study entry, the part A CFZ group had higher Cryptosporidium shedding, total stool weight, and more diarrheal episodes compared with the placebo group. Over the inpatient period, compared with those who received placebo, the CFZ group Cryptosporidium shedding increased by 2.17 log2 Cryptosporidium per gram stool (95% upper confidence limit, 3.82), total stool weight decreased by 45.3 g (P = .37), and number of diarrheal episodes increased by 2.32 (P = .87). The most frequent solicited adverse effects were diarrhea, abdominal pain, and malaise. One placebo and 3 CFZ participants died during the study. Plasma levels of CFZ in participants with cryptosporidiosis were 2-fold lower than in part B controls.

**Conclusions:**

Our findings do not support the efficacy of CFZ for the treatment of cryptosporidiosis in a severely immunocompromised HIV population. However, this trial demonstrates a pathway to assess the therapeutic potential of drugs for cryptosporidiosis treatment. Screening persons living with HIV for diarrhea, and especially Cryptosporidium infection, may identify those failing ARV therapy.

**Clinical Trials Registration:**

NCT03341767.

## Teaser

We evaluated clofazimine for treatment of adults living with human immunodeficiency virus and cryptosporidiosis. Clofazimine was well tolerated but did not reduce *Cryptosporidium* excretion or diarrhea compared with adults treated with placebo. This trial forms a blueprint for future cryptosporidiosis therapeutic trials.

*Cryptosporidium* infection and diarrhea (cryptosporidiosis) is a life-threatening in persons living with human immunodeficiency virus (HIV) and also in young children in the developing world [1]. In children, cryptosporidiosis causes severe diarrhea [2], malabsorption and intestinal injury [3], excess mortality [2, 4], and stunting and is associated with malnutrition [5]. There is a huge unmet need for *Cryptosporidium* drugs [6]; only nitazoxanide is licensed for treatment of cryptosporidiosis, but it has not shown any benefits as a treatment for immunocompromised patients living with HIV and cryptosporidiosis compared with placebo [7–9].

Clofazimine (CFZ), which has been used to treat leprosy for more than 50 years and currently is part of the treatment for multidrug-resistant tuberculosis (TB), has recently been described as effective against *Cryptosporidium* in vitro [10]. The efficacy and pharmacokinetics (PK) of CFZ in patients living with HIV and cryptosporidiosis are not known. We developed an experimental medicine study design to evaluate the safety, tolerability, PK, and efficacy of CFZ in adults living with HIV and cryptosporidiosis.

## METHODS

### Study Design and Participants

The study was a single-center, randomized, double-blind, placebo-controlled phase 2a 2-part study at Queen Elizabeth Central Hospital in Blantyre, Malawi. Participants were eligible for part A if they were living with HIV, aged 18–65 years, weighed more than 35.4 kg, were on antiretrovirals (ARV) for at least 1 month, and had diarrhea duration of a minimum of 14 days. Participants for part B were living with HIV without diarrhea or *Cryptosporidium* and met none of the exclusion criteria. Full criteria are listed in the Supplementary Appendix. The study protocol was approved by the relevant regulatory and ethics committees before study initiation [11]. Participants provided written informed consent.

### Study Treatment and Procedures

**Figure 1.**
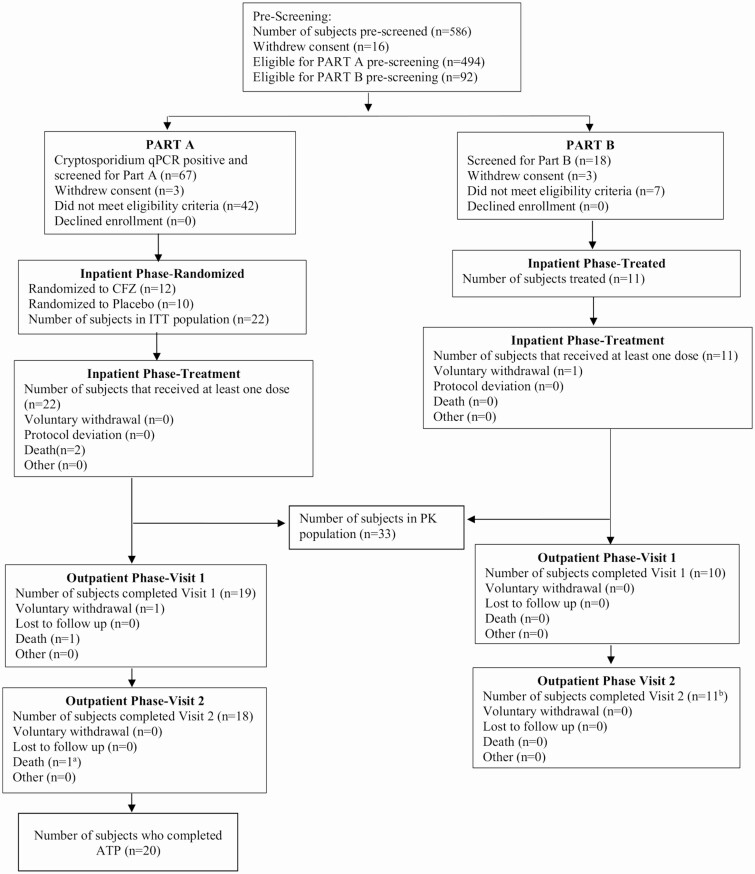
Part A trial profile. ^a^Participant died after completing visit. ^b^One participant withdrew during the inpatient phase but provided final blood draw. Abbreviations: ATP, according to protocol; CFZ, clofazimine; ITT, intention to treat; PK, pharmacokinetics; qPCR, quantitative polymerase chain reaction.

Part A participants were randomized 1:1 to receive either 5 days of oral CFZ or placebo (Figure 1). The dosage of CFZ administered was the maximum given in clinical practice, 100 mg 3 times daily for participants who weighed ≥50 kg or 50 mg 3 times daily for participants who weighed <50 kg [12]. Participants for part B were matched 1:1 to the first 10 part A participants based on age (±5 years), gender, and weight (≥50 kg or <50 kg; Supplementary Appendix).

We used a rapid diagnostic test (RDT) for *Cryptosporidium* screening (prototype immunochromatographic test strip for detecting *Cryptosporidium*; TechLabs Inc, Blacksburg, VA) and an enzyme-linked immunosorbent assay (ELISA) stool test (CRYPTOSPORIDIUM II, TechLabs Inc) for assessing *Cryptosporidium* shedding in serial stools during the trial. All *Cryptosporidium* shedding was confirmed and measured using quantitative polymerase chain reaction (qPCR), with a positive result being a cycle threshold (Ct) <35. The first collected stool of the day was obtained throughout the dosing and follow-up periods for testing of the *Cryptosporidium* ELISA signal, as well as for measurement of *Cryptosporidium* shedding by qPCR. In addition, all stool samples were collected and pooled in 8-hour intervals during the inpatient phase of the study, days –1 to 5 of dosing. Thus, total *Cryptosporidium* stool excretion was measured using qPCR during this time.

Stool enteropathogens present at baseline in addition to *Cryptosporidium* were detected using qPCR in a TaqMan Array Card (Thermo Fisher, Waltham, MA) using a custom design developed at the Houpt Laboratory (Charlottesville, VA; Supplementary Appendix) [13]. Measurements of antiretroviral (ARV) levels in plasma and alteration after administration of CFZ were evaluated at the Van Voorhis/Arnold Laboratories (Seattle, WA). CFZ concentrations in plasma and stool were measured at Q_2_ Solutions (Ithaca, NY).

After the 5-day inpatient study drug dosing, with daily clinical examination and laboratory sampling, all participants entered a 2-month follow-up period that included a visit 19–24 days post last dose and a final visit 41–55 days post last dose. During each visit and with weekly phone calls, participants were monitored for safety and symptoms. Safety laboratory tests were repeated if there were any abnormalities previously. If participants could not be reached by phone, home visits were made.

### Outcomes

There were 2 primary end points for part A: the first was efficacy, assessed as a reduction in the (log) number of *Cryptosporidium* shed in the first collected stool of each study dosing day from CFZ vs placebo recipients in participants treated according to protocol (ATP). The second primary end point was safety, including frequency and severity of solicited and unsolicited adverse events (AEs), serious adverse events (SAEs), AEs of special interest, and suspected, unexpected serious adverse reactions. Part B had 2 primary end points (CFZ in plasma and total daily amount of CFZ eliminated in stool) to meet a single primary PK objective. Secondary end points were the reduction in the (log_2_) number of *Cryptosporidium* shed in stool compared with controls in the intention-to-treat (ITT) population, reduction in total daily *Cryptosporidium* shedding in those treated ATP and compared with controls in the ITT population, and reduction in severity of diarrhea over the study dosing period compared with controls.

An independent data safety monitoring board (DSMB) was involved in regular review of blinded safety data to monitor risks and benefits and to assess any potential safety issues that might arise during the study. Trial site monitoring of participant safety was carried out by the sponsor medical monitor, an independent local safety monitor, and the contract research organization medical monitor and was overseen by the chief investigator (W. V. V.). 

### Statistical Analyses

As the phase 2a study was exploratory, we initially planned an interim analysis after 20 participants were randomized and treated ATP. This sample size was predicted to detect a therapeutic difference based on animal data from molecular end points. Due to slow enrollment, we decided to convert the interim analysis to a final analysis (Supplementary Appendix).

The primary ATP analysis was performed using the randomized population who received at least 80% of scheduled doses, completed daily assessments of fecal shedding, and had no major protocol deviations. When missing data for the primary end point (log number of *Cryptosporidium* shed per gram stool) were not attributable to nondetectable *Cryptosporidium* (ie, no stooling), multiple imputation was used (Supplementary Appendix).

The safety population consisted of all participants who received at least 1 dose of the study drug. The PK population consisted of all participants who had at least 1 measurable PK concentration (Supplementary Appendix).

Due to the exploratory nature of the trial, no adjustments due to multiple testing were made. All statistical tests were performed with a 1-sided alpha of 0.05. Statistical analyses were conducted using SAS version 9.3.

## RESULTS

Between 18 December 2017 and 14 February 2019, 5790 adults were approached to assess eligibility. For randomization to CFZ vs placebo (part A), 494 were prescreened for *Cryptosporidium* presence in stool via RDT and qPCR, 67 participants were *Cryptosporidium* PCR-positive in stool and screened, and 22 were randomized (12 to CFZ and 10 to placebo, ITT group; Figure 1). Twenty participants completed inpatient dosing ATP. There was 1 voluntary withdrawal (CFZ group) during the outpatient phase. There was no loss to follow-up.

The RDT and ELISA stool test had low sensitivity (41% for both) to identify participants and follow the presence/absence of *Cryptosporidium* over time compared with qPCR. The *Cryptosporidium* spp. identified were *C. parvum* (11/22, 50%), *C. meleagridis* (4/22, 18%), *C. hominis (*3/22, 14%), *C. viatorum* (1/22, 5%), and 3 unknowns. Coinfection of stool with multiple diarrhea enteropathogens was common, with a median of 4 copathogens (excluding *Cryptosporidium*) per participant (range, 1–8). The most frequently identified copathogen was enteroaggregative *Escherichia coli* (64%), followed by *Shigella* toxin-positive enterotoxigenic *E. coli* (41%) and *Shigella*/enteroinvasive *E. coli* (23%). The baseline characteristics of participants are listed in Table 1. Despite randomization, compared with the placebo group, the CFZ group had by chance more males (67% vs 20%), lower body mass index (16.3 ± 1.7 vs 18.0 ± 3.1 kg/m^2^), increased diarrhea output total stool weight (320.3 ± 214.6 vs 245.8 ± 299.4 g), more pathogens detected at a diarrheagenic amount per Global Enteric Multicenter Study (GEMS) criteria (67% vs 30%) [14], more advanced HIV immunosuppression (CD4 counts 25.3 ± 24.4 vs 170.4 ± 321.7 cells/µL), and higher prevalence of *C. parvum* detected (58% vs 40%).

**Table 1.** Baseline Characteristics of Participants

Characteristic	Part A CFZ Group (n = 12)	Part A Placebo Group (n = 10)	Part B CFZ Group (n = 11)
Age, y	39.8 (± 7.8)	39.1 (± 12.0)	44.1 (± 9.6)
Male sex (%)	8 (67)	2 (20)	7 (64)
Body mass index, kg/m^2^	16.3 (± 1.7)	18.0 (± 3.1)	18.9 (± 1.4)
Pulse rate, beats/min	90.9 (± 12.4)	95.9 (± 14.9)	78.1 (± 6.7)
Systolic blood pressure, mm Hg	99.3 (± 15.0)	106.4 (± 16.5)	116.5 (± 11.8)
Diastolic blood pressure, mm Hg	68.3 (± 10.1)	71.2 (± 8.8)	75.7 (± 12.3)
Hemoglobin, g/dL	10.6 (± 2.2)	10.8 (± 2.8)	14.0 (± 1.3)
Hematocrit, %	32.3 (± 6.5)	32.6 (± 8.7)	42.3 (± 3.6)
White blood cells, 10^9^/L	2.9 (± 1.4)	3.8 (± 2.8)	5.0 (± 1.7)
Neutrophils, 10^9^/L	1.6 (± 0.9)	2.1 (± 2.1)	2.5 (± (± 1.2)
Lymphocytes, 10^9^/L	0.8 (± 0.5)	1.1 (± 0.7)	2.0 (± 0.8)
CD4 absolute, cells/µL			
Mean (± standard deviation)	25.3 (± 24.4)	170.4 (± 321.7)	422.0 (± 231.3)
Median (interquartile range)	23.0 (8.0–32.0)	22.5 (17.0–86.0)	361.0 (216.0–634.0)
Human immunodeficiency virus viral load, copies/µL	241 981.5 (± 262 806.03)	679 025.13 (± 929 116.49)	257.5 (± 805.7)
Antiretroviral therapy duration, days	1424 (± 1547.6)	2011 (± 1409.3)	1265 (±1810.3)
Blood urea nitrogen, mmol/L	4.9 (± 2.5)	3.9 (± 1.1)	3.8 (± 1.0)
Creatinine, µmol/L	82.0 (± 37.2)	56.0 (± 15.9)	65.4 (± 14.0)
Alanine aminotransferase, IU/L	34.0 (± 20.3)	40.3 (± 19.5)	38.9 (± 21.4)
Aspartate aminotransferase, IU/L	50.6 (± 16.4)	63.0 (± 30.4)	50.7 (± 18.3)
Electrocardiogram			
Normal (%)	11 (92)	10 (100)	11 (100)
Abnormal, not clinically significant (%)	1 (8)	0 (0)	0 (0)
QTc interval, ms	421.7 (± 14.2)	418.3 (± 17.0)	409.7 (± 21.6)
*Cryptosporidium* spp. (%)			
* C. parvum*	7 (58%)	4 (40%)	N/A
*C. hominis*	2 (17%)	1 (10%)	N/A
*C. meleagridis*	1 (8%)	3 (30%)	N/A
*C. viatorum*	1 (8%)	0 (0%)	N/A
Unknown^a^	1 (8%)	2 (20%)	N/A
Copathogens detected at diarrheagenic amount (%)	8 (67)	3 (30)	N/A
Diarrhea duration,^b^ days	17 (± 7.6)	34 (± 57)	N/A
Stool enzyme-linked immunosorbent assay positivity (D-1, %)	7 (58)	2 (20)	N/A
Log number of *Cryptosporidium* shed in first collected stool of day, *Cryptosporidium* per gram stool (D-1)	13.9 (± 2.7)	15.0 (± 2.2)	N/A
Total daily *Cryptosporidium* shedding, *Cryptosporidium* per gram stool (D-1)	22.3 (± 2.9)	22.1 (± 3.2)	N/A
Total stool weight, g (D-1)	320.3 (± 214.6)	245.8 (± 299.4)	N/A
Most severe diarrhea severity grade^c^ (mild)	9 (75%)	3 (30%)	N/A
Stool consistency severity grade ≥ 3 (D-1, %)	9 (75)	6 (67)	N/A
Number of diarrheal episodes,^c^ D1	1.3 (± 1.1)	0.8 (± 1.3)	N/A

All values are mean (± standard deviation), unless otherwise listed.

Abbreviations: CFZ, clofazimine; D, day; N/A, not available.

^a^Failed to amplify on sequencing of 18s and gp60.

^b^Participants with diarrhea duration entries “ >2 weeks” were treated as 21 days for calculations of summary statistics.

^c^Observed over the first 24-hour dosing interval after the first study dose.

**Figure 2.**
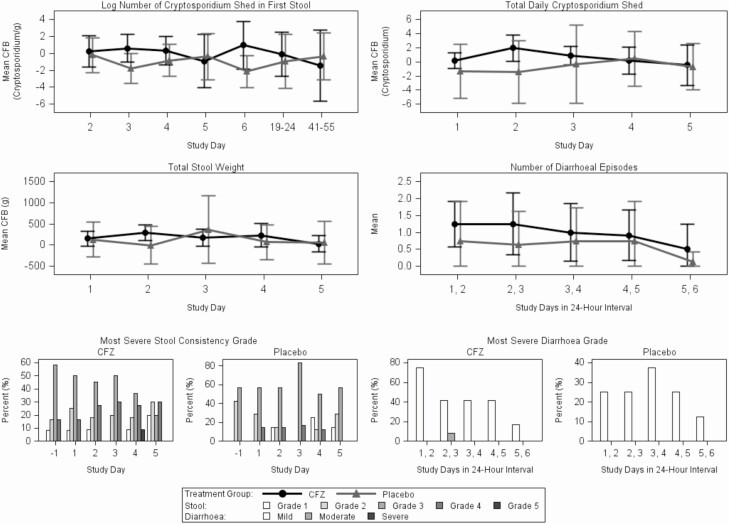
Treatment response in the according-to-protocol group. *A,* Mean CFB in log number of *Cryptosporidium* shed in first collected stool over time. *B,* Mean CFB in total daily *Cryptosporidium* shedding over time. *C,* Mean CFB in total stool weight over time. *D,* Mean number of diarrheal episodes over time. *E,* Proportion of most severe stool consistency grade by time. *F,* Proportion of most severe diarrhea grade by time. Abbreviations: CFB, change from baseline; CFZ, clofazimine.

Findings were similar for both ATP and ITT populations (Supplementary Table 1), and the ATP efficacy results are reported here. Stool *Cryptosporidium* excretion was persistent among part A participants throughout observation (Supplementary Figures 1 and 2), even at 41–55 days after the last dose. There was no significant difference in *Cryptosporidium* shedding in the CFZ group compared with placebo (Figure 2A and 2B). There was a trend toward increased change from baseline in *Cryptosporidium* shedding in the first stool of the day in the CFZ-treated group vs placebo, with a difference in means of 2.17 log_2_*Cryptosporidium* per gram (95% upper confidence limit [CL], 3.82]) and in total *Cryptosporidium* shedding with a difference in means of 1.02 log_2_*Cryptosporidium* (95% upper CL, 2.50); this result is opposite to what is expected if CFZ was efficacious. There was no significant change in diarrhea in the CFZ group compared with placebo, whether measured by total stool weight change from baseline, number of diarrheal episodes, stool consistency grade, or severity diarrhea grade (Figure 2C–2F).

**Figure 3.**
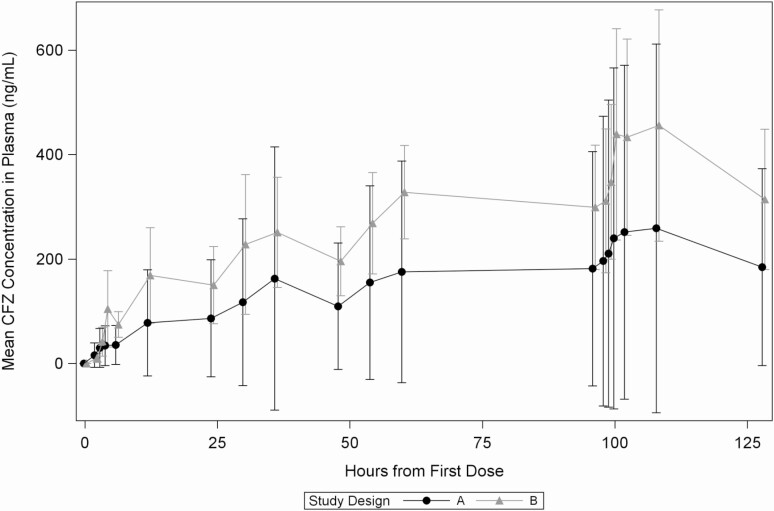
Mean plasma concentration of CFZ in plasma by time. Abbreviation: CFZ, clofazimine.

For the PK of CFZ in participants living with HIV without diarrhea or *Cryptosporidium* (part B), 92 were prescreened, 18 were screened, and 11 received CFZ, with 1 voluntary withdrawal during the inpatient phase. Part A participants had about 2-fold less plasma exposure of CFZ than part B participants on day 5 (ratio area under the curve [AUC]_0–24_, 0.607) and on day 1 of the inpatient dosing (ratio AUC_0–24_, 0.478; Table 2, Figure 3; stool PK profiles are listed in the Supplementary Appendix and Supplementary Figure 3).

**Table 2.** Summary of Adverse Events

Type of Adverse Event	Level of Severity	Part A Clofazimine (n =12) (%)	Part A Placebo (n = 10) (%)	Part B (n = 11) (%)
Any solicited AE	Any severity	12 (100)	10 (100)	0 (0)
	Max severity	2 (17)	0 (0)	0 (0)
Abdominal pain	Any severity	8 (67)	7 (70)	0 (0)
	Max severity	1 (8)	0 (0)	0 (0)
Vomiting	Any severity	4 (33)	4 (40)	0 (0)
	Max severity	1 (8)	0 (0)	0 (0)
Diarrhea	Any severity	9 (75)	4 (40)	0 (0)
	Max severity	0 (0)	0 (0)	0 (0)
Anorexia	Any severity	4 (33)	3 (30)	0 (0)
	Max severity	0 (0)	0 (0)	0 (0)
Skin discoloration	Any severity	0 (0)	0 (0)	0 (0)
Nausea	Any severity	5 (42)	5 (50)	0 (0)
	Max severity	1 (8)	0 (0)	0 (0)
Malaise	Any severity	6 (50)	3 (30)	0 (0)
	Max severity	1 (8)	0 (0)	0 (0)
Urgency of defecation	Any severity	5 (42)	4 (40)	0 (0)
	Max severity	0 (0)	0 (0)	0 (0)
Any AEs with fatal outcome		3 (25)	1 (10)	0 (0)
Number of unsolicited AEs		13	12	3
Participants with ≥1 unsolicited AE		6 (50)	4 (40)	3 (27)
Participants with a serious AE		5 (42)	2 (20)	0 (0)
Any unsolicited AE related to study drug		2 (17)	0 (0)	3 (27)
Any unsolicited AE leading to discontinuation of study drug		0 (0)	1 (10)	0 (0)

Abbreviation: AE, adverse event.

For safety, solicited AEs (Table 3), which are expected in persons with diarrhea, were experienced by all participants in both CFZ and placebo groups. There were higher numbers of solicited AEs experienced in the CFZ group for diarrhea (9, 75% vs 4, 40% in placebo), abdominal pain (8, 67% vs 7, 70% in placebo), and malaise (6, 50% vs 3, 30% in placebo) and more severe solicited AEs in the CFZ group (2, 17%) than the placebo group (0, 0%; Supplementary Figures 4 and 5). No part B participants experienced any solicited AEs. The number of unsolicited AEs (Supplementary Table 2) was highest in the CFZ group (13 vs 12 in placebo and 3 in part B); the number of participants who experienced AEs with a fatal outcome was also higher in the CFZ group (3, 25% vs 1, 10% in placebo and none in part B). None of the fatalities were judged by the study medical monitors and DSMB to be CFZ-related (Supplementary Appendix).

**Table 3.** Comparison of Pharmacokinetic Parameters in Part A and Part B Participants

Pharmacokinetics Parameter		Part A (n = 12)		Part B (n = 11)	
		Mean (± SD)	% CV	Mean (± SD)	% CV
Day 1	C_min_ (ng/mL)	35.83 (± 37.28)	323	74.74 (± 24.51)	46
	C_max_ (ng/mL)	97.55 (± 117.9)	195	193.3 (± 93.50)	58
	T_max_ (h)	19.73 (± 5.67)	–	14.776 (± 7.537)	–
	AUC_0-24_ (ng × h/mL)	1364.0 (± 1754.0)	219	2851.0 (± 1256.0)	50
Day 5	C_min_ (ng/mL)	258.8 (± 353.1)	187	455.8 (± 221.5)	47
	C_max_ (ng/mL)	280.7 (± 355.2)	173	514.1 (± 202.0)	39
	T_max_ (h)	9.679 (± 10.81)	–	6.683 (± 3.765)	–
	AUC_0-24_ (ng × h/mL)	6863.0 (± 8552.0)	172	11 298.0 (± 5580.0)	59
Summary	t_1/2_ (h)^a^	336.5 (± 84.71)	25	535.5 (± 4.950)	1
	R_AUC_	5.905 (± 3.516)	57	4.111 (± 1.579)	50

Abbreviations: AUC, area under the curve; C_max_, peak plasma concentration; C_min_, trough plasma concentration; CV, coefficient of variation; R_AUC_, accumulation ratio for AUC_0-24_ for day 5 to day 1; SD, standard deviation; t_1/2_, elimination half-life; T_max_, time to reach C_max_.

^a^Elimination half-life of clofazimine was previously found to be ≤70 days upon repeat dose administration; therefore, the relatively short plasma sampling schedule in this study may not accurately capture the t_1/2_ parameter in these populations.

## DISCUSSION

This is the first randomized, double-blind, placebo-controlled phase 2a trial to evaluate CFZ for treatment of cryptosporidiosis in adults living with HIV. The trial demonstrated that CFZ had no significant impact on *Cryptosporidium* shedding of the parasite or on diarrheal episodes, stool weight, and consistency compared with placebo. Evaluation of *Cryptosporidium* shedding in the first stool of the day provided similar data to total daily *Cryptosporidium* shedding. The drug is generally well tolerated. Four patients died, 3 of whom received CFZ and the fourth placebo. This rate of death was consistent with our a priori estimates, and each case was reviewed by the independent DSMB. CFZ achieved 2-fold less plasma exposure among part A participants with diarrhea vs part B participants without diarrhea.

The trial did show that adults living with HIV with ≥3 days of diarrhea consistently excreted *Cryptosporidium* in their stools, even when assayed up to 60 days after enrollment. This demonstrates that this population would be appropriate to study the antiparasitic benefit of anti-*Cryptosporidium* drugs that do not depend on the immune response.

The trial did not show a reduction in *Cryptosporidium* excretion in this population treated with CFZ vs placebo. This was the case whether one compared the *Cryptosporidium* excretion by qPCR, as determined by the concentration in the first stool of the day, or by determining the total *Cryptosporidium* excreted per day. In fact, there was a nonsignificant trend toward slightly increased *Cryptosporidium* shedding in the CFZ group vs the placebo, which was most evident at day 2 of study drug dosing. The trend toward increased shedding may reflect the more ill status of the CFZ participants at baseline, as documented in their enrollment laboratory results and health status. With a median HIV CD4 count of 23.5 cells/mm^3^ (interquartile range [IQR], 11.75–43.75) and viral load of 168 097.5 copies/mL (IQR, 94 044–643 812.3), the mortality rate of 18% in the trial likely reflects advanced disease in our part A cohort as a whole.

Within our cohort, compared with placebo, the CFZ group had more deaths, SAEs, and severe solicited AEs. All participants with cryptosporidiosis reported the solicited AEs expected with CFZ, such as diarrhea, abdominal pain, malaise, and nausea. However, these solicited AEs were present at baseline in part A participants, as might be expected in this population with cryptosporidiosis, and were universal in both treatment groups. There tended to be fewer solicited AEs over time, which correlated with less severity in diarrhea during the hospital phase, and the severity of AEs tended to decrease over time. None of the part B participants exposed to the same dose of CFZ reported solicited AEs, and only 3 part B participants reported unsolicited AEs, and these were generally mild.

A previous clinical trial for cryptosporidiosis treatment identified multiple safety concerns related to the health status of participants. That phase 1–2 trial of miltefosine to treat HIV-related cryptosporidiosis in Zambian adults with chronic diarrhea was terminated early due to high mortality, lack of efficacy, and development of SAEs that were attributed to the extreme metabolic abnormalities already present in patients enrolled in the trial [16]. In our trial, participants with cryptosporidiosis also presented with electrolyte abnormalities, most commonly hypokalemia that required correction, and some required corrective treatment through the trial. In addition, there was also a very high incidence of active TB in the screening population living with HIV. Screening by chest X-ray was inadequate likely because dehydrated participants often do not have an infiltrate until rehydrated. Screening of sputum using GeneXpert or Gram stain also was inadequate due to the inability of dehydrated participants to produce sputum. All deaths in our study were reported prior to instituting urine lipoarabinomannan (LAM) screening at baseline. Once urine LAM screening was instituted [17], 43% of our otherwise eligible participants subsequently tested positive by urine LAM and were excluded.

Part A participants were extremely immunosuppressed. Most had CD4 counts <25 cells/µL and high HIV viral loads. Plasma levels of HIV medicines were detected at similar levels to part B participants (unpublished data), suggesting that these part A participants were compliant with first-line ARV therapy and that ARV resistance might be driving HIV treatment failure. Therefore, screening for diarrhea in this population, especially for *Cryptosporidium*, delineated those more at risk for TB and ARV failure.

The predominant *Cryptosporidium* species was *C. parvum* subtype family IIc anthroponotic (10/11, 91% of those with *C. parvum*). This was unexpected, given that the commonly identified *Cryptosporidium* species in the pediatric GEMS and adult studies was *C. hominis* [18–21]. However, a high prevalence of *C. parvum* has been noted in HIV/AIDS patients in Ethiopia, where 92/140 (66%) of HIV/AIDS patients were positive by PCR-restricted fragment length polymorphism (RFLP) [22]. As *C. parvum* has been associated with prolonged diarrhea in persons living with HIV more frequently than *C. hominis* [18], the trial inclusion criteria may have selected for this species.

Multiple copathogens were observed in stool, which may have contributed to the diarrhea [3]; however, patients with symptomatic diarrhea were routinely treated with ciprofloxacin as standard of care. *Cryptosporidium* may have driven the diarrhea in at least 15 of 22 participants in this trial, as it was the pathogen with the lowest C_t_ value and may have been the pathogen in the greatest quantity shed in stool. After applying GEMS cutoffs, which use C_t_ counts to determine clinically relevant diarrhea [14], only 7 *Cryptosporidium* samples met diarrheagenic cutoffs, and only 11 samples met diarrheagenic pathogen criteria. As GEMS data were based on children, the lack of correlation between C_t_ value and clinical diarrhea likely reflects the differences seen in an adult population with severe HIV immunosuppression with prolonged diarrhea.

Our PK data suggest that diarrhea and/or *Cryptosporidium* infection negatively impact CFZ plasma exposure. Since efficacy is likely driven by CFZ levels in the parasite, which may not be well exposed to intraluminal CFZ as it is located in a vacuole under the epithelial plasma membrane and faces in toward the gut lumen [23], plasma levels may not reflect efficacy as it would for systemic infections. The fact that serum CFZ levels in persons with well-suppressed HIV were twice as high suggests that in the setting of *Cryptosporidium* infection, the drug was not well absorbed. We propose that lower levels of CFZ likely existed in the epithelium layer in the part A participants, as passage through the gastrointestinal epithelium is required for access to the plasma. These lower levels may have contributed to the failure of efficacy against *Cryptosporidium*. However, we used the maximum dosage of CFZ that is accepted as safe in this trial [12]; therefore, increasing the dosage to improve efficacy may not be feasible. An intravenous form of clofazimine, described in the past [24], may have provided better systemic delivery of the drug; however, this was not a formulation available at the time of the trial.

One limitation of the study was the small sample size. This led to slightly uneven randomization (12 vs 10) based on block size. Also, imbalances in the baseline characteristics were noted in the part A CFZ vs placebo groups, with the CFZ group being more ill at baseline. One possible confounder was the presence of multiple copathogens in the stool, which could have influenced diarrhea resolution.

For the conduct of future human experimental trials of cryptosporidiosis in this population, this study suggests that the screening population be evaluated for TB detection through urine LAM and for electrolyte disturbances, particularly hypokalemia; that the use of stool RDT in screening and ELISA tests on serial stools is not as sensitive as qPCR and that we need only use qPCR to enroll and follow participants for *Cryptosporidium* excretion over time; that following serial *Cryptosporidium* shedding by qPCR of the first stool of the day, rather than total stool collection, is probably sufficient to assess efficacy; that given the ill status of enrolled participants, an inpatient trial is merited, and AEs and deaths may complicate safety evaluation of new study drugs; and that future trials would need to be multisite given the slow recruitment rate. Until this trial, there were few placebo-controlled trials in adults [25–27] and limited data on how to test the drugs in phase 2a. This trial shows that adults living with HIV and cryptosporidiosis excrete the parasite consistently and thus that the effects of treatment on excretion would be a feasible way to monitor for efficacy in *Cryptosporidium* therapy.

In conclusion, this is the first controlled clinical trial to assess the safety, efficacy, and PK of CFZ for treatment of cryptosporidiosis. Although CFZ does not show promise as a novel therapeutic for *Cryptosporidium* infection, future human studies can use an approach based on lessons learned in this trial to assess the therapeutic potential of drugs for treatment of cryptosporidiosis.

## SUPPLEMENTARY DATA

Supplementary materials are available at *Clinical Infectious Diseases* online. Consisting of data provided by the authors to benefit the reader, the posted materials are not copyedited and are the sole responsibility of the authors, so questions or comments should be addressed to the corresponding author.

## Supplementary Material

ciaa421_suppl_Supplementary_Appendix

## Notes

***Acknowledgments.*** The authors thank the participants who took part in this study. We thank the Cryptofaz study team members, including administrative, clinical, laboratory, pharmacy, data, and ancillary staff in Malawi and Liverpool School of Tropical Medicine; and the Emmes CC-ID8 team in Maryland and their site monitor team in India (Pankaj Dua, Anand Singh, Abhishek Kumar). We thank the Queen Elizabeth Central Hospital management and Blantyre district health office for granting us permission to use their health facilities; the medical monitors (Frederick Buckner and Jamie Rylance); the Data Safety Monitoring Board (Steven Reynolds [chair], David Boulware, Jane Mallewa, David Lalloo, and Maia Lesosky); Bill & Melinda Gates Foundation (BMGF) program officers for valuable discussions and advice; Brigitte Denis and George Selemani for Malawi-Liverpool Wellcome Trust Clinical Research Programme (MLW) laboratory support; Clemens Masesa for MLW data management support; Sarah Burke and Q2 Solutions for determining clofazimine levels in plasma and stool; Leonardo Sahelijo for facilitating the site initiation visit; Joel Herbein and TechLabs for donating rapid diagnostic and enzyme-linked immunosorbent assay (ELISA) tests for *Cryptosporidium* testing; James Platts-Mills and Jie Liu for assistance with TaqMan Array Card studies in Houpt Lab; and Claire Colson, Janice Yu, and Mikasa Morf for University of Washington administrative support.

***Financial support***. The work was supported by the BGMF (OPP1172544). Novartis provided both the clofazimine and placebo. TechLabs provided *Cryptosporidium* rapid diagnostic and ELISA tests.

***Potential conflicts of interest.*** P. I., K. J., M. L., C. M., and W. V. V. have received grants from the BMGF, outside of the submitted work. M. L. and C. M. have received a supplemental grant from BMGF for preclinical and early clinical development of clofazimine as a treatment for cryptosporidiosis (OPP1156296). C. M. and M. L. report grants OPP1107194 and OPP1208899 from BMGF. K. J. is a Wellcome International Training Fellow (grant 201945/Z/16/Z) and has received investigator-initiated grant support from the GlaxoSmithKline Biologicals group of companies. D. Hermann is a current employee of BMGF. W. V. V. has patents issued for bumped kinase inhibitors (BKIs) for the therapeutic treatment of cryptosporidiosis diarrhea and is a founder of and has stock in ParaTheraTech LLC, a company that is developing BKIs for animal health indications. All other authors report no potential conflicts. All authors have submitted the ICMJE Form for Disclosure of Potential Conflicts of Interest. Conflicts that the editors consider relevant to the content of the manuscript have been disclosed.
