# Supplementary material for: Clofazimine for Treatment of Cryptosporidiosis in Human Immunodeficiency Virus Infected Adults: An Experimental Medicine, Randomized, Double-blind, Placebo-controlled Phase 2a Trial
Source: Clin Infect Dis. 2020 Apr 11;73(2):183–91. doi: 10.1093/cid/ciaa421 (PMC8282326; doi:10.1093/cid/ciaa421)

Supplementary Appendix

Table of contents

1. **Methods**
   1. Study design
   2. Participants
   3. Randomization and masking
   4. Procedures
   5. Outcomes
   6. Statistical analyses
   7. Role of the funding source
2. **Results**

2.1 Stool PK profile

2.2 Fatal outcomes

1. References
2. **Supplementary Table 1.** Efficacy of clofazimine compared to placebo in the according to protocol (ATP) and intention-to-treat (ITT) populations
3. **Supplementary Table 2.** Total number of unsolicited adverse events
4. **Supplementary Figure 1.** Treatment response in the intention-to-treat group:
5. Mean change from baseline (CFB) in log_2_ number of cryptosporidium shed in first collected stool over time
6. Mean change from baseline (CFB) in total daily cryptosporidium shedding over time
7. Mean total daily cryptosporidium shedding over time
8. Mean change from baseline (CFB) in total stool weight over time
9. Mean number of diarrheal episodes over time
10. Proportion of most severe stool consistency grade by time
11. Proportion of most severe diarrhea grade by time
12. **Supplementary Figure 2**. Stool cryptosporidium shedding in:
13. First stool of the day
14. Total daily stooling
15. **Supplementary Figure 3.** Mean amount of CFZ in stool by timepoint
16. **Supplementary Figure 4.** Maximum severity of solicited symptoms
17. **Supplementary Figure 5.** Frequency of adverse events by organ class and:
18. Severity
19. Relationship to treatment
20. **Methods**

1.1 Study design

The study was a single center, randomized, double-blind, placebo-controlled Phase 2a two-part study at Queen Elizabeth Central Hospital in Blantyre, Malawi. Subjects were screened at this government, tertiary-level hospital, which serves the Southern region of the country, and were also referred from surrounding health centers within the Blantyre district. The study protocol and relevant supporting materials were approved by the National Health Sciences Research Committee (NHSRC) and the Pharmacy, Medicines, and Poisons Board in Malawi, and the Liverpool School of Tropical Medicine research ethics committee before study initiation.^1^ Participants provided written informed consent. The NHSRC set participant compensation levels were used.

1.2 Participants

Participants were eligible for Part A if they met the following inclusion criteria: HIV-infected, aged 18-65 years, weight over 35·4 kg, on antiretrovirals (ARV) for at least 1 month, and with diarrhea duration of minimum 14 days. We estimated a priori a death rate in the HIV population in Malawi to be approximately 15%. Recruitment commenced on 18 December, 2017. On 6 April, 2018 after five subjects were randomized, eligibility criteria were amended to include participants with diarrhea duration of a minimum of 3 days and who have been on ARV for a minimum two weeks. Criteria were amended due to slow recruitment. Exclusion criteria included fever; evidence of active tuberculosis (by chest x-ray, sputum positive for TB by GeneXpert or Acid Fast Bacilli, and after 13 subjects were randomized, positive urine lipoarabinomannan (LAM)); history of allergy or hypersensitivity to CFZ; significant cardiac arrhythmia or ECG abnormalities; history of additional risk factors for Torsade de Pointes; family history of long QT syndrome; use of concomitant medications that markedly prolong the QT interval; pregnant and lactating women; use of systemic corticosteroids or anti-*Cryptosporidial* treatments within the preceding 28 days; and subjects with clinically significant laboratory value abnormalities at screening (hemoglobin <5 g/dL, serum potassium <3·0 mEq/L, and aspartate aminotransferase (AST) or alanine transaminase (ALT) ≥3 times upper limit of normal).

Participants for Part B were HIV-infected without diarrhea or *Cryptosporidium*, met none of the exclusion criteria, and were matched 1:1 to the first ten Part A subjects based on age (±5 years), gender, and weight (≥ or <50 kg).

1.3 Randomization and masking

We used a computer-generated randomization schedule where Part A group assignments of CFZ (Lamprene®, Novartis, Switzerland) and placebo were allocated in a 1:1 ratio, respectively, using a permuted block design with block size 4. Randomization was done by a contracted third-party contract research organization (CRO, Emmes, Rockville, MD, USA) that were involved in oversight but not the day-to-day clinical management of the study. The study drug and placebo were identical in appearance. Only the Emmes statisticians conducting the analysis and the pharmacists who prepared the pill packs were unmasked. The investigators, participants, and study site personnel involved in treating and assessing participants were masked to treatment allocation until the data was locked to further changes.

1.4 Procedures

Enrolled participants received five days of oral CFZ 50 mg three times daily for subjects <50 kg, or 100 mg three times daily if ≥50 kg, or placebo, respectively. Each dose was given half an hour after consumption of a fortified peanut-based paste (Plumpy Nut®, Nutriset, France). Participants were hospitalized for the five days of the study drug administration and returned on site for two follow-up visits. Laboratory testing was primarily carried out on-site at the Malawi-Liverpool Wellcome Trust Clinical Research Programme (MLW) laboratories. We used a rapid diagnostic test (RDT) for *Cryptosporidium* screening (prototype immunochromatographic test strip for detecting *Cryptosporidium*, TechLabs Inc., Blacksburg, VA, USA) and an ELISA stool test (CRYPTOSPORIDIUM II™, TechLabs Inc.) for quantifying *Cryptosporidium* shedding in serial stools during the trial. All *Cryptosporidium* shedding was confirmed and measured by qPCR, with a positive result being a cycle threshold (Ct) <35. The first collected stool of the day was obtained throughout the dosing and follow-up periods, for testing of the *Cryptosporidium* ELISA signal, as well as for measurement of *Cryptosporidium* shedding by qPCR. In addition, all stools were collected and pooled in 8-hour intervals during the inpatient phase of the study, Days -1 to 5 of dosing. Thus, total *Cryptosporidium* stool excretion was measured by qPCR during this time.

Stool enteropathogens present at baseline in addition to *Cryptosporidium* were detected using qPCR in a TaqMan Array Card (TAC, Thermo Fisher, Waltham, MA, USA) using a custom design developed at the Houpt Laboratory (Charlottesville, VA, USA).^2^ TAC assays were performed at MLW, and also included previously published qPCR assays that distinguished *C. hominis* and *C. parvum*.^3^ Further characterization of *Cryptosporidium* from baseline samples was achieved using Sanger sequencing targeting the 18S^4^ and gp60 genes^5^ performed at the Houpt Laboratory. The primer pairings originally described in Glaberman et al.^5^ for the amplification of gp60 prior to Sanger sequencing were modified such that 5’-ATAGTCTCCGCTGTATTC-3’ was paired with 5’-GGAAGGAACGATGTATCT-3’ for the primary amplification and 5’-TCCGCTGTATTCTCAGCC-3’ was paired with 5’-GCAGAGGAACCAGCATC-3’ for the secondary nested amplification. Measurements of ARV levels in plasma and alteration after administration of CFZ were evaluated in the Van Voorhis/Arnold Laboratories (Seattle, WA, USA). Measurement of CFZ concentration in plasma and stool were performed at Q_2_ Solutions (Ithaca, NY, USA) using liquid chromatography-tandem mass spectrometry (LC/MS/MS), which were validated for quantification of CFZ within the range of 1·0-1000 ng/mL in human plasma.

After study drug dosing, all participants entered a follow-up period of two months that included a follow-up visit within 19-24 days post last dose, and a final visit 41-55 days post last dose. During each follow-up visit and with weekly phone calls, participants were monitored for safety and symptoms. Blood and stool specimens were collected at each visit, and safety labs were repeated if there were any abnormalities previously. If participants could not be reached by phone, home visits were made.

In a resource-limited setting such as Malawi, laboratory investigations are not always available and therefore clinical care is primarily reliant on clinical presentation and symptoms. As part of the clinical care, laboratory results were reviewed by a clinician and subjects were referred for additional care as needed.

1.5 Outcomes

There were two primary endpoints for Part A, though formal statistical testing was only utilized for the primary efficacy endpoint. The first primary endpoint was efficacy, assessed as reduction in the (log) number of *Cryptosporidium* shed in the first collected stool of each study dosing day of CFZ vs. placebo recipients in subjects treated according to protocol (ATP). The second primary endpoint was safety, based on safety assessments collected throughout dosing and follow-up periods, and consisted of frequency and severity of solicited and unsolicited adverse events (AEs) through study product administration, including serious adverse events (SAEs), adverse events of special interest (AESIs) and suspected, unexpected serious adverse reactions (SUSARs). Part B had two primary endpoints (CFZ in plasma, and total daily amount of CFZ eliminated in stool) to meet a single primary PK objective.

Secondary endpoints were the reduction in the (log_2_) number of *Cryptosporidium* shed in stool compared to controls in the intention-to-treat (ITT) population, reduction in total daily *Cryptosporidium* shedding in those treated ATP, and as compared to controls in the ITT population, and reduction in severity of diarrhea over the study dosing period compared to controls.

1.6 Statistical analyses

Calf data on fecal shedding over time (unpublished data, Michael Riggs, University of Arizona) suggested that 10 individuals treated in each arm would be sufficient to give a >80% chance of seeing a difference with an efficacious drug. We were uncertain about the relevance of the animal data to the HIV subjects, and whether they would have consistent shedding over the period of treatment. Thus, we arbitrarily increased the sample size to 28 per group.

As the Phase 2a study was exploratory, we initially planned an interim analysis after 20 subjects were randomized and treated ATP. Due to slow enrollment, it was decided to convert the interim analysis to a final analysis.

Efficacy endpoints for Part A were summarized descriptively, and continuous efficacy variables were summarized at baseline and in terms of change from baseline at each day following study drug administration. The primary ATP analysis was performed using the randomized population who received at least 80% of scheduled doses, completed daily assessments of fecal shedding, and had no major protocol deviations. When missing data for the primary endpoint (log number of *Cryptosporidium* shed per gram stool) was not attributable to non-detectable *Cryptosporidium* (i.e. no stooling), multiple imputation was utilized. The fully conditional specification (FCS) method for arbitrary longitudinal missing data patterns was used to perform multiple imputation of the missing primary efficacy variable as well as any missing covariates. Mixed ANCOVA models for repeated measures were used to model and analyze the difference, between treatment groups, in the change from baseline in continuous endpoints over the inpatient period, and generally included baseline response, day, and treatment group as covariates. Gender and age were also included as covariates in models for the log number of cryptosporidium shed in the first collected stool (analyzed in the ATP and ITT populations). The day by treatment interaction term was considered for inclusion in models if statistically significant, and if included, the difference in efficacy measures in the last inpatient day was reported. Proportional odds models were used for the analysis of categorical endpoints (e.g., stool consistency and diarrhea severity). Upper 95% confidence intervals (CI) and p-values were derived from each model.

The safety population consisted of all subjects that received at least one dose of study drug. All safety analyses were descriptive. Ten subjects enrolled in Part B were matched to the first 10 subjects who completed Part A ATP, to develop a comparative description of the absorption and excretion of the drug in the two groups. The PK population consisted of all subjects who had at least one measurable PK concentration. Plasma and stool drug concentrations were plotted at each timepoint for matched Part A and Part B subjects together, on linear scale. PK parameters were estimated through a non-compartmental analysis using Phoenix WinNonlin version 8.0 or later (Pharsight Corporation, Cary, NC, USA). The paired t-test was used to assess differences between groups for each PK parameter on days 1 and 5 (C_min_, C_max_, and AUC_0-24_), and reported the geometric mean ratio between groups. We reported the Hodges-Lehmann estimator (pseudomedian) for the difference in each parameter between Part A and Part B subjects. P-values and 95% CI for each of the above tests were calculated.

Due to the exploratory nature of the trial, no adjustments due to multiple testing were made; all statistical tests were performed with a one-sided alpha of 0.05. Statistical analyses were conducted using SAS version 9.3.

To maximize the safety and integrity of the study, an independent data safety monitoring board (DSMB) was involved in regular review of blinded safety data to monitor risks and benefits and to assess any potential safety issues arising during the study. Trial site monitoring of participant safety was carried out by the sponsor medical monitor, an independent local safety monitor, the CRO medical monitor, and overseen by the chief investigator (WVV). This study is registered with ClinicalTrials.gov, number NCT03341767.

1.7 Role of the funding source

The funders of the study had no role in study design, data collection, data analysis, data interpretation, or writing of the report. The first and last authors (PI, WVV) and the funders had full access to all the data in the study, following data lock. The first and last authors were responsible for the decision to submit for publication.

1. **Results**

2.1 Stool PK profiles

The total observed daily amount of CFZ eliminated in the feces on days 2 and 5 was not significantly different between Part A and B subjects (Supplementary Figure 3). Less than 2% of the cumulative CFZ doses was recovered in stool in both groups over the five days of stool collection.

2.2 Fatal outcomes

Two CFZ-treated subjects developed a fatal sepsis-like syndrome on day 5 of dosing. The first, judged to be unrelated to study drug, developed severe fatigue on the morning of the last dosing day with documented hypotension and was judged to have sepsis, received ceftriaxone and intravenous fluids, but rapidly died despite therapy. The second fatal case occurred in a subject who developed abdominal pains a day after receipt of CFZ, resolved when CFZ was stopped, then recurred when CFZ was restarted. An abdominal ultrasound demonstrated biliary stones, but a surgical consult could not be organized before the subject died of sepsis-like syndrome. The site judged the death to be related to CFZ administration, although the study medical monitors and DSMB judged this fatal SAE to be unrelated. The third CFZ-treated subject that died presented to the hospital with profound hypotension and diarrhea on day 18 after receipt of study drug. The patient did not respond to fluid resuscitation in the emergency suite and expired quickly after arrival. Death was attributed to the effects of chronic diarrhea, AIDS, and delayed presentation. The fatal SAE in the placebo group occurred 47 days after study drug administration, in a subject who had been diagnosed with pulmonary and extrapulmonary TB after randomization with rehydration. The latter two deaths were judged not to be related to treatment. No autopsies were conducted in any of the deaths so exact causes of death could not be ascribed.

1. **References**

1. Nachipo P, Hermann D, Quinnan G, Gordon MA, Van Voorhis WC, Iroh Tam PY. Evaluating the safety, tolerability, pharmacokinetics and efficacy of clofazimine in cryptosporidiosis (CRYPTOFAZ): study protocol for a randomized controlled trial. *Trials* 2018; **19**(1): 456.

2. Liu J, Kabir F, Manneh J, et al. Development and assessment of molecular diagnostic tests for 15 enteropathogens causing childhood diarrhoea: a multicentre study. *Lancet Infect Dis* 2014; **14**(8): 716-24.

3. Hadfield SJ, Robinson G, Elwin K, Chalmers RM. Detection and differentiation of Cryptosporidium spp. in human clinical samples by use of real-time PCR. *J Clin Microbiol* 2011; **49**(3): 918-24.

4. Sow SO, Muhsen K, Nasrin D, et al. The Burden of Cryptosporidium Diarrheal Disease among Children < 24 Months of Age in Moderate/High Mortality Regions of Sub-Saharan Africa and South Asia, Utilizing Data from the Global Enteric Multicenter Study (GEMS). *PLoS Negl Trop Dis* 2016; **10**(5): e0004729.

5. Glaberman S, Moore JE, Lowery CJ, et al. Three drinking-water-associated cryptosporidiosis outbreaks, Northern Ireland. *Emerg Infect Dis* 2002; **8**(6): 631-3.

**Supplementary Table 1.** Efficacy of clofazimine compared to placebo in the according to protocol (ATP) and intention-to-treat (ITT) populations

| Outcomes | Difference in means | 95% upper confidence limit | P-value |
| --- | --- | --- | --- |
| **Parasitologic** | | | |
| Change from baseline in log_2_ number of cryptosporidium shed in first collected stool (log_2_ *Cryptosporidium* per gram), ATP | 2·17 | 3·82 | 0·984 |
| Change from baseline in log_2_ number of cryptosporidium shed in first collected stool (log_2_ *Cryptosporidium* per gram), ITT | 1·73 | 3·13 | 0·977 |
| Change from baseline in total daily cryptosporidium shedding (log_2_ *Cryptosporidium*), ATP | 1·02 | 2·50 | 0·877 |
| Change from baseline in total daily cryptosporidium shedding (log_2_ *Cryptosporidium*), ITT | 0·16 | 1·69 | 0·569 |
| **Diarrheal** | | | |
| Change from baseline in total stool weight at Day 5 (g), ATP | 132·05 | 314·48 | 0·888 |
| Change from baseline in total stool weight at Day 5 (g), ITT | -45·30 | 179·88 | 0·366 |
| Number of diarrheal episodes, ATP | 1·92 | 5·73 | 0·802 |
| Number of diarrheal episodes, ITT | 2·32 | 5·74 | 0·871 |
| **Characteristics** | Odds ratio |  |  |
| Most severe stool consistency grade, ATP | 0·66 | 10·39 | 0·401 |
| Most severe stool consistency grade, ITT | 1·85 | 26·26 | 0·651 |
| Most severe diarrhea grade, ATP | 5·33 | 29·28 | 0·947 |
| Most severe diarrhea grade, ITT | 4·87 | 23·85 | 0·950 |

**Supplementary Table 2.** Total number of unsolicited adverse events

|  |  | Part A – CFZ (n=12) | Part A – placebo (n=10) | Part B (n=11) |
| --- | --- | --- | --- | --- |
| MedDRA® system organ class | MedDRA® preferred term | No. of events | No. of events | No. of events |
| Any system organ class | Any preferred term | 13 | 12 | 3 |
| Blood and lymphatic system disorders | Anemia | 0 | 3 | 0 |
| Gastrointestinal disorders | Any preferred term | 4 | 0 | 0 |
|  | Abdominal pain | 1 | 0 | 0 |
|  | Anal fissure | 1 | 0 | 0 |
|  | Diarrhea | 2 | 0 | 0 |
| General disorders and administration site conditions | Pyrexia | 0 | 1 | 0 |
| Infections and infestations | Any preferred term | 4 | 6 | 0 |
|  | Extrapulmonary tuberculosis | 0 | 1 | 0 |
|  | Gastroenteritis | 1 | 1 | 0 |
|  | Lower respiratory tract infection | 1 | 0 | 0 |
|  | Esophageal candidiasis | 0 | 1 | 0 |
|  | Oral candidiasis | 0 | 1 | 0 |
|  | Pneumonia | 0 | 1 | 0 |
|  | Pulmonary tuberculosis | 0 | 1 | 0 |
|  | Sepsis | 1 | 0 | 0 |
|  | Septic shock | 1 | 0 | 0 |
| Investigations | Any preferred term | 1 | 0 | 3 |
|  | Alanine aminotransferase increased | 0 | 0 | 2 |
|  | Neutrophil count decreased | 0 | 0 | 1 |
|  | White blood cell count decreased | 1 | 0 | 0 |
| Metabolism and nutrition disorders | Hypokalemia | 1 | 1 | 0 |
| Skin and subcutaneous tissue disorders | Decubitus ulcer | 1 | 0 | 0 |
| Vascular disorders | Any preferred term | 2 | 1 | 0 |
|  | Hypotension | 1 | 1 | 0 |
|  | Hypovolemic shock | 1 | 0 | 0 |

CFZ, clofazimine; MedDRA®, medical dictionary for regulatory activities

**Supplementary Figure 1.** Treatment response in the intention-to-treat group:

1. Mean change from baseline (CFB) in log_2_ number of cryptosporidium shed in first collected stool over time


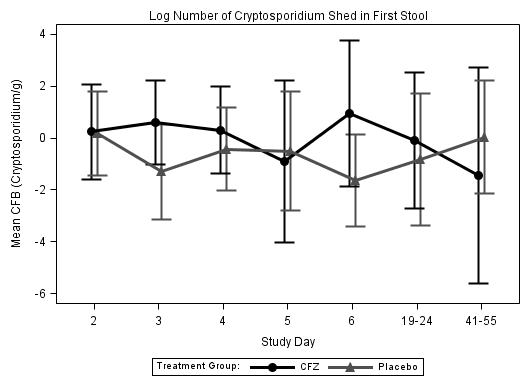


1. Mean CFB in total daily cryptosporidium shedding over time


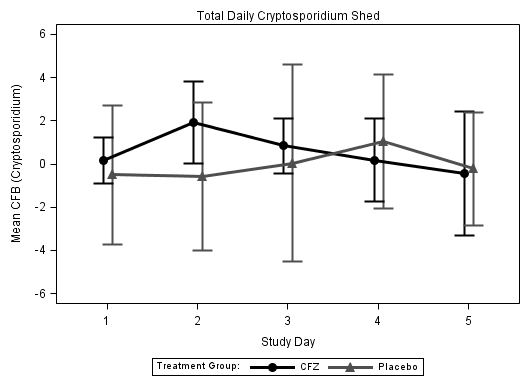


1. Mean total daily cryptosporidium shedding over time


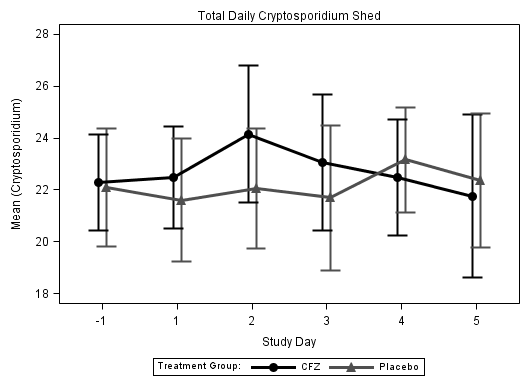


1. Mean CFB in total stool weight over time


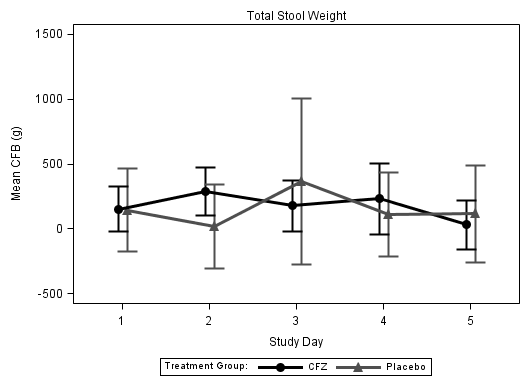


1. Mean number of diarrheal episodes over time


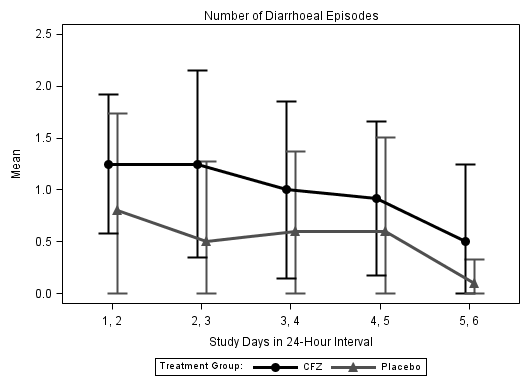


1. Proportion of most severe stool consistency grade by time


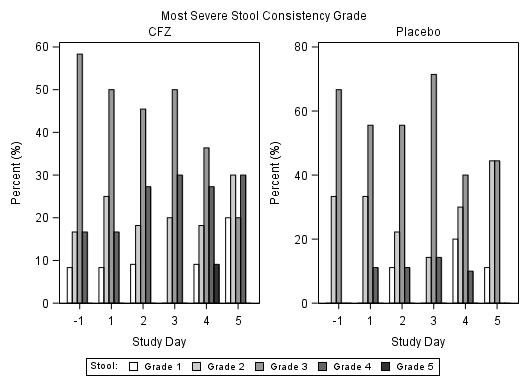


1. Proportion of most severe diarrhea grade by time


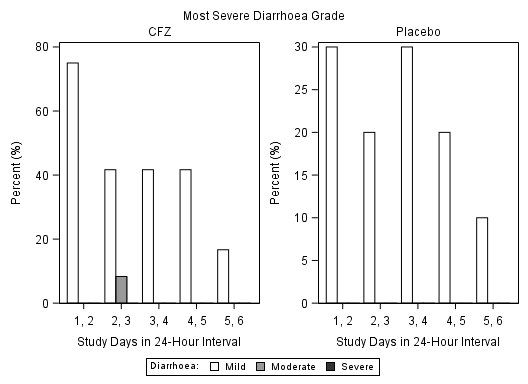


**Supplementary Figure 2**. Stool cryptosporidium shedding in:

1. First stool of the day


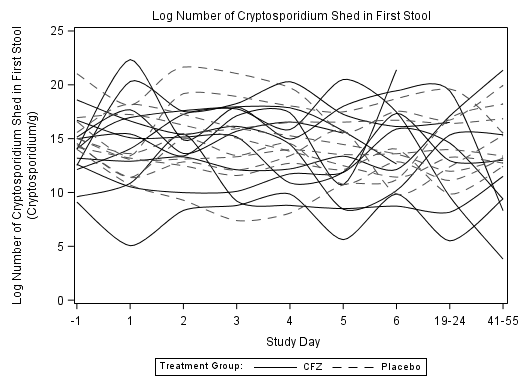


1. Total daily stooling


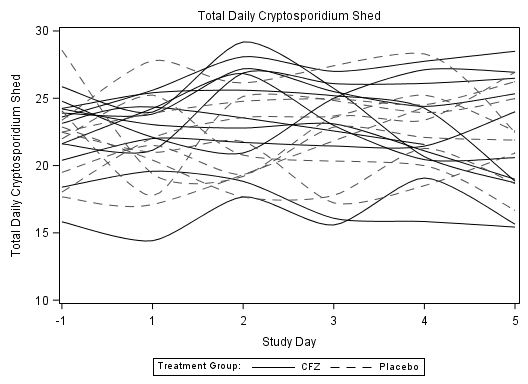


**Supplementary Figure 3.** Mean amount of CFZ in stool by timepoint


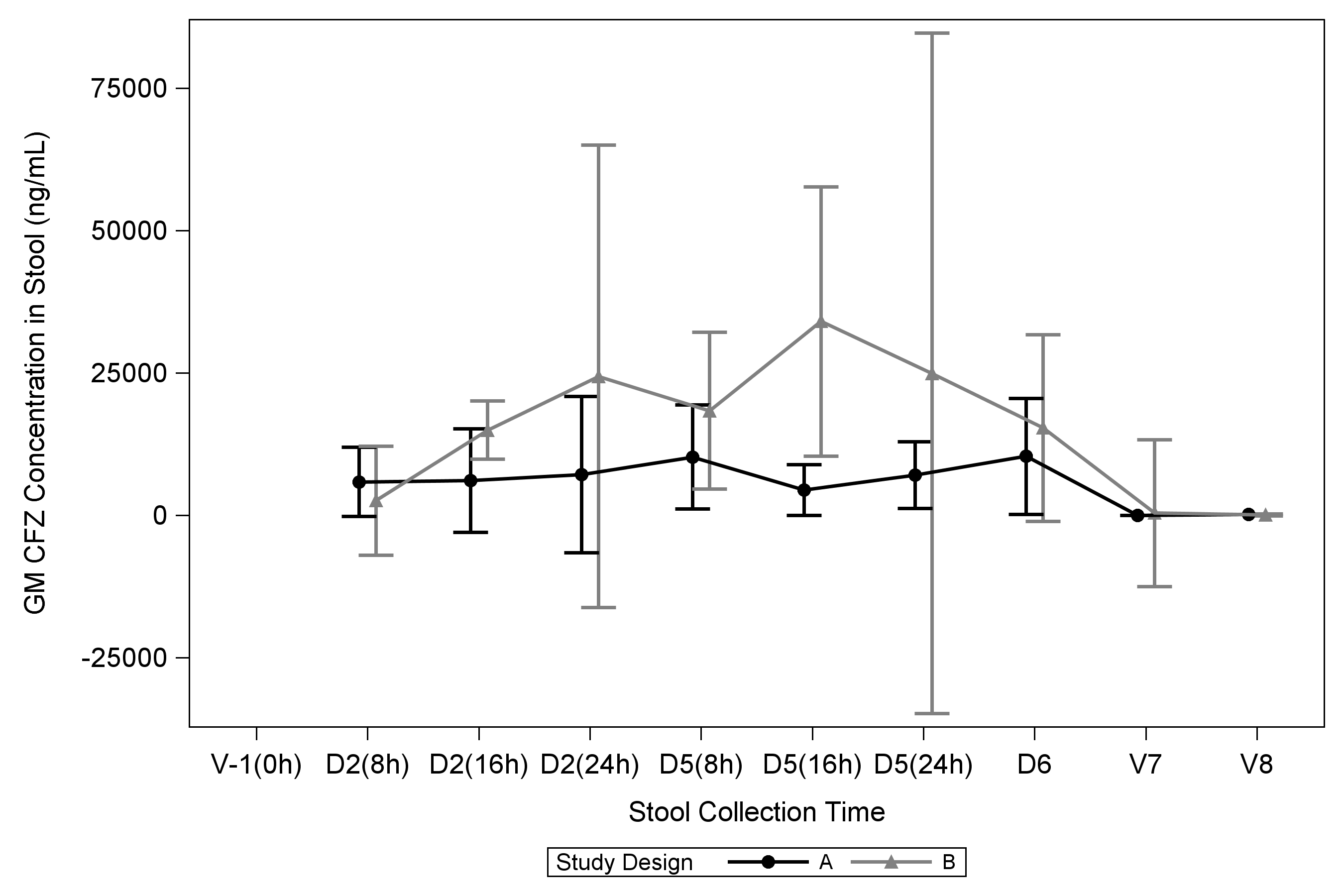


CFZ, clofazimine; D, day; GM, geometric mean; V, study visit

**Supplementary Figure 4.** Maximum severity of solicited symptoms


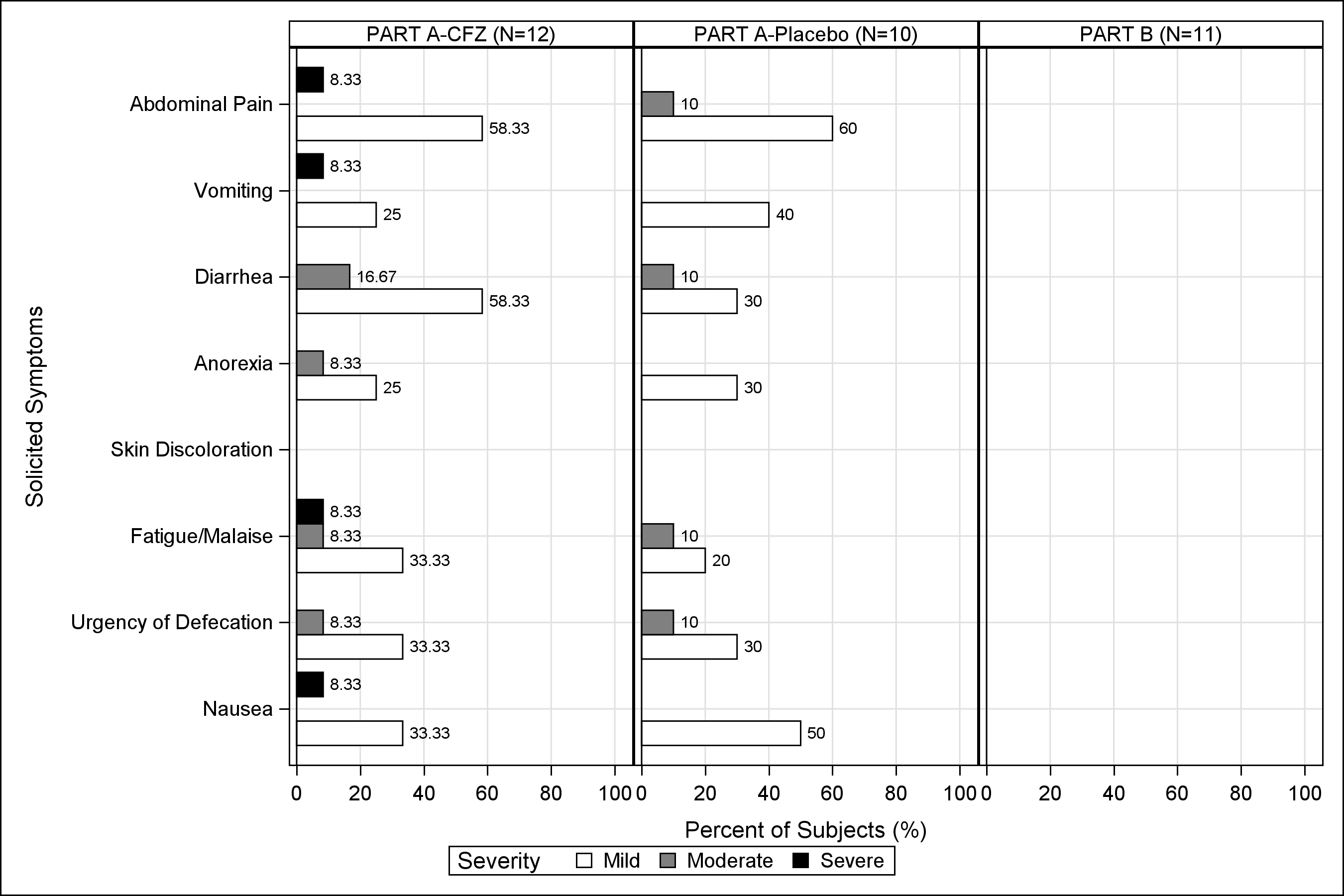


**Supplementary Figure 5.** Frequency of adverse events by organ class and:

1. Severity


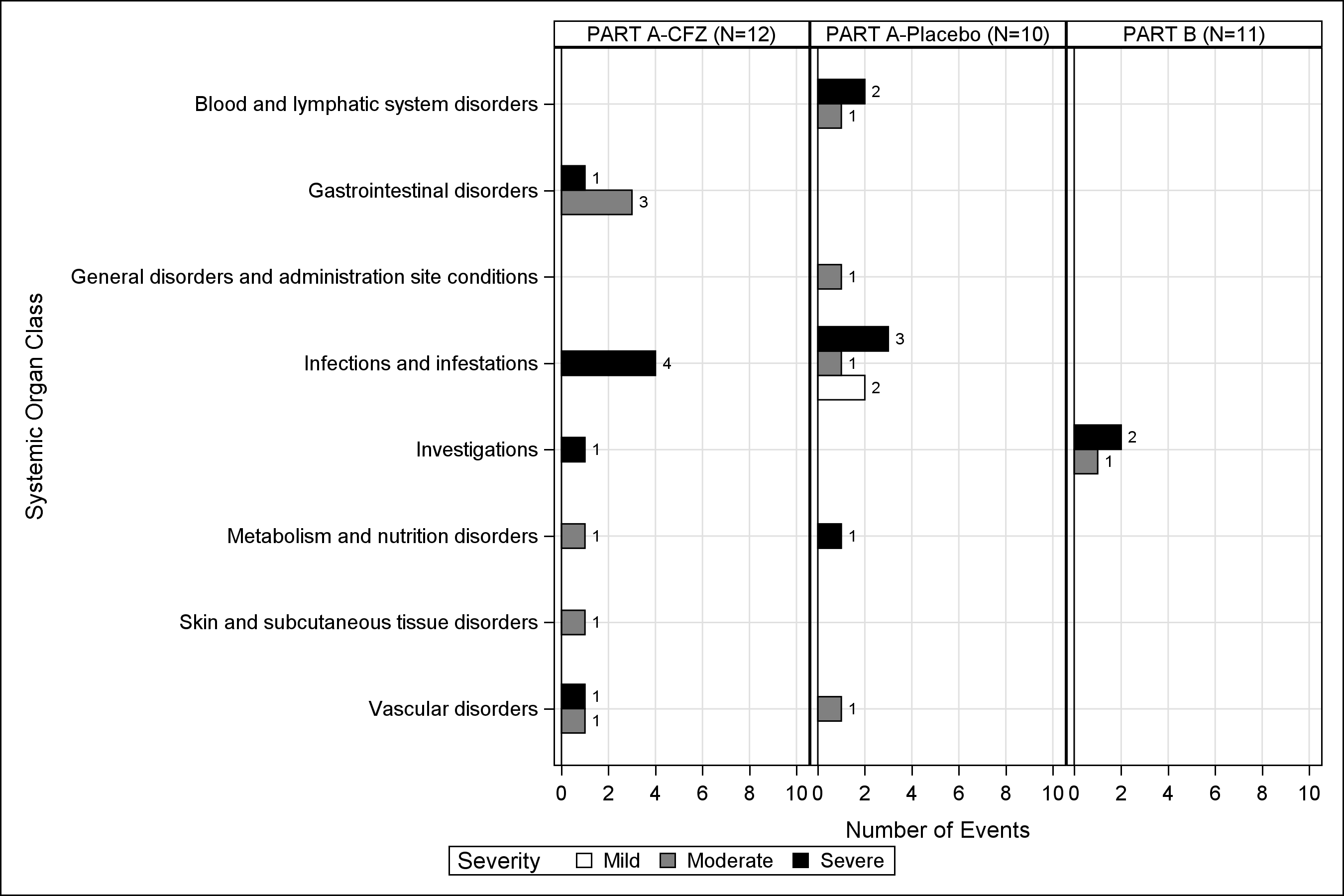


1. Relationship to treatment


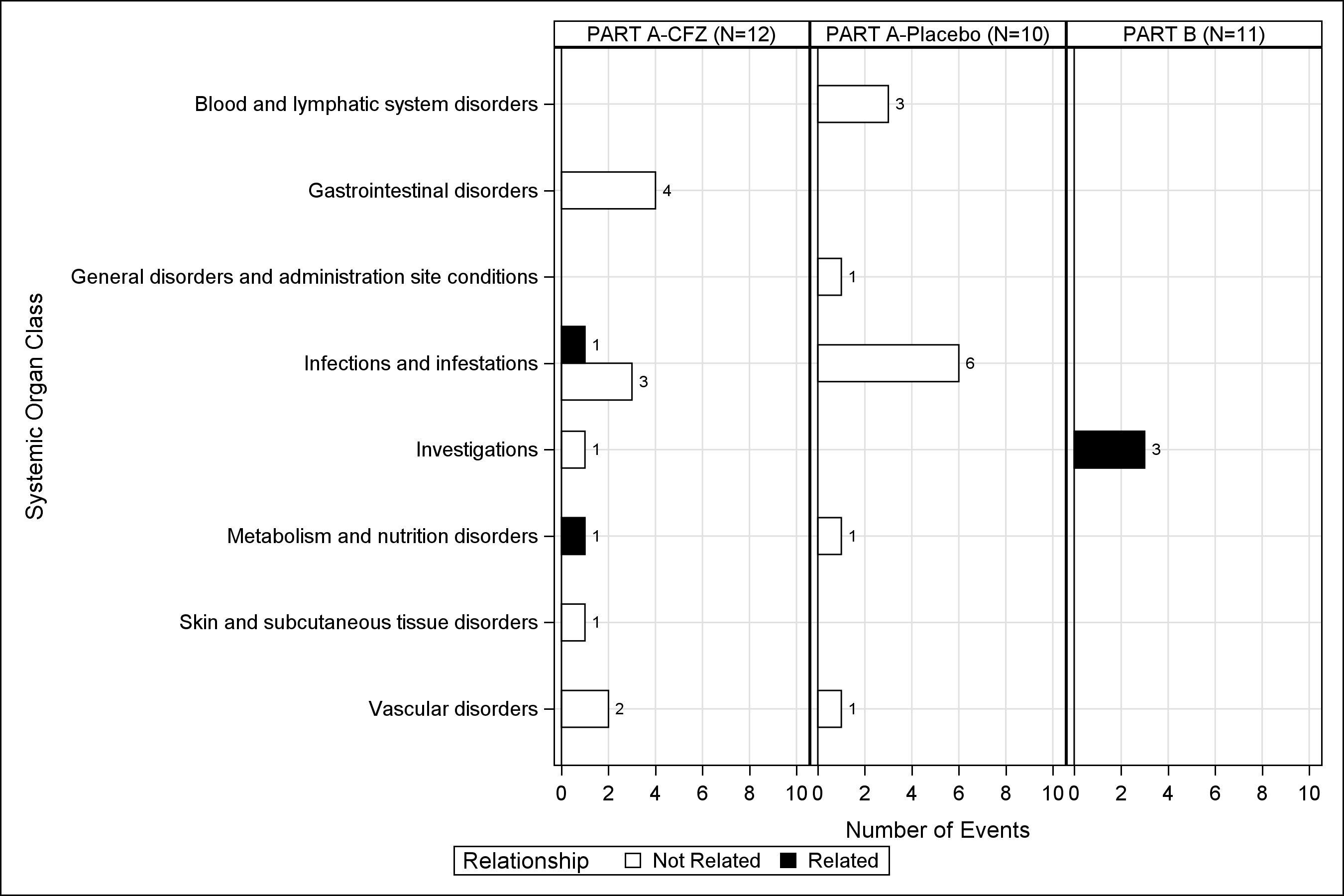

Supplement: ciaa421_suppl_Supplementary_Appendix [file ciaa421_suppl_supplementary_appendix.doc]
